# Differential proteomics analysis of the analgesic effect of electroacupuncture intervention in the hippocampus following neuropathic pain in rats

**DOI:** 10.1186/1472-6882-12-241

**Published:** 2012-12-02

**Authors:** Yong-Hui Gao, Shu-Ping Chen, Jun-Ying Wang, Li-Na Qiao, Fan-Ying Meng, Qiu-Ling Xu, Jun-Ling Liu

**Affiliations:** 1 Department of Physiology, Institute of Acupuncture and Moxibustion, China Academy of Chinese Medical Sciences, Beijing, 100700, China

**Keywords:** Acupuncture analgesia, Cumulative effect, Hippocampus, Chronic constrictive injury

## Abstract

**Background:**

Evidence is building steadily on the effectiveness of acupuncture therapy in pain relief and repeated acupuncture-induced pain relief is accompanied by improvement of hippocampal neural synaptic plasticity. To further test the cellular and molecular changes underlying analgesic effect of acupuncture, the global change of acupuncture associated protein profiles in the hippocampus under neuropathic pain condition was profiled.

**Methods:**

The chronic constrictive injury (CCI) model was established by ligature of the unilateral sciatic nerve in adult Wistar rats. Rats were randomized into normal control (NC) group, CCI group, and CCI with electroacupuncture (EA) stimulation group. EA was applied to bilateral Zusanli (ST36) and Yanglingquan (GB34) in the EA group. Differentially expressed proteins in the hippocampus in the three groups were identified by two-dimensional gel electrophoresis and matrix-assisted laser desorption/ionization time of flight mass spectrometry. The functional clustering of the identified proteins was analyzed by Mascot software.

**Results:**

After CCI, the thermal pain threshold of the affected hind footpad was decreased and was reversed gradually by 12 sessions of acupuncture treatment. Following EA, there were 19 hippocampal proteins identified with significant changes in expression (>2-fold), which are involved in metabolic, physiological, and cellular processes. The top three canonical pathways identified were “cysteine metabolism”, “valine, leucine, and isoleucine degradation” and “mitogen-activated protein kinase (MAPK) signaling”.

**Conclusions:**

These data suggest that the analgesic effect of EA is mediated by regulation of hippocampal proteins related to amino acid metabolism and activation of the MAPK signaling pathway.

## Background

Low back pain, including sciatica, is a common symptom that causes severe psychological and economical problems [1-3]. It is well established that acupuncture therapy is effective in relieving symptoms of patients with neuropathic pain [4-6]. Experimental studies have also demonstrated that acupuncture intervention can effectively relieve chronic neuropathic and inflammatory pain in rats [7,8]. Hence, acupuncture therapy is considered a better choice for pain relief due to the limited options, side effects, and insufficient analgesia of currently available drugs.

The mechanism underlying acupuncture analgesia is believed to relate to induction of functional responses in various brain regions. Animal studies have identified a number of mesencephalic and brainstem nuclei putatively involved in mediating acupuncture information [9-11]. Interestingly, fMRI studies [12-14] showed that acupuncture stimulation resulted in activation or deactivation of multiple limbic areas including the hippocampal complex. We previously reported that repeated acupuncture intervention-induced pain relief in rats with chronic constrictive injury (CCI) of the sciatic nerve was accompanied by improvement of hippocampal neural synaptic plasticity and regulation of CaMKII and synaptophysin expression [15].

Numerous studies have demonstrated a close association between the hippocampal activity and neuropathic pain [16-19]. Alterations in the morphology and gene expression in the hippocampus have been detected under different pain conditions [20]. Correspondingly, multiple proteins or peptides [20-23], including galanin, rat spinal cord expression protein 1, and NF-kappa B, were reported to play key roles in neuropathic pain. However, at present there are limited protein network data linking the hippocampus to the acupuncture analgesic effect. Furthermore, as the cellular and molecular changes underlying cumulative acupuncture analgesia are very complicated, identification of new proteins involved in this process will help elucidate the underlying mechanism of acupuncture analgesia.

In the present study, we examined the global changes of acupuncture intervention-associated proteins in the hippocampus and identified the protein function clusters and potential signaling pathways in CCI rats. The use of two-dimensional gel electrophoresis (2-DE) and matrix-assisted laser desorption/ionization time of flight mass spectrometry (MALDI-TOF MS) approach enabled us to perform an unbiased and comprehensive analysis of protein expression changes [24].

## Methods

### Animals

A total of 60 female Wistar rats (240–300 g) were obtained from the Experimental Animal Center of the Peking Union Medical College (Beijing) and housed within the animal care facilities in the Institute of Acupuncture and Moxibustion, China Academy of Chinese Medical Sciences. Animals were randomized into normal control (NC) (sham ligature), CCI model, and CCI with electroacupuncture (EA) stimulation groups (n = 20 per group). Ten animals in each group were used for protein extraction and 10 for RNA extraction. All experimental procedures were approved by the Institute of Acupuncture and Moxibustion of China Academy of Chinese Medical Sciences and were identical to the “*Guidelines for Laboratory Animal Care and Use*” of the Chinese Ministry of Science and Technology (2006).

### CCI Pain model and pain threshold detection

The CCI model was established by ligating the unilateral sciatic nerve, as previously reported [25,26]. Briefly, under anesthesia (25% urethane plus 2x1.5% chloralose, 0.4 mL/100 g body weight) and routine sterilization, the left sciatic nerve was exposed at the mid-thigh level by blunt dissection through the biceps femoris. Four constrictive ligatures (4–0 non-absorbable suture) were tied around the nerve at the distal end close to the bifurcation site at approximately 1 mm spacing. The ligature was alright until a moderate muscular contraction of the leg could be seen. For the NC group, the rats underwent the same procedure but without nerve ligature.

Five days after CCI, and every three days after CCI, the withdrawal latency (PWL; i.e., thermal pain threshold) of the bilateral hind paws was determined using a 37370 Algesia Detector (Ugo, Italy). The radiant heat source was focused on the plantar surface of a hindpaw, and a light intensity was preset to obtain a baseline latency of approximately 15 s. A cut-off time was set at 20 s to avoid tissue damage. Each rat underwent two trials with a 5 min interval, and the mean value of two trials was used as the withdrawal latency. To minimize differences in individual animals, the difference value of PWL (PWLD) between the healthy and the affected side was calculated.

### Electroacupuncture

**Figure 1:**
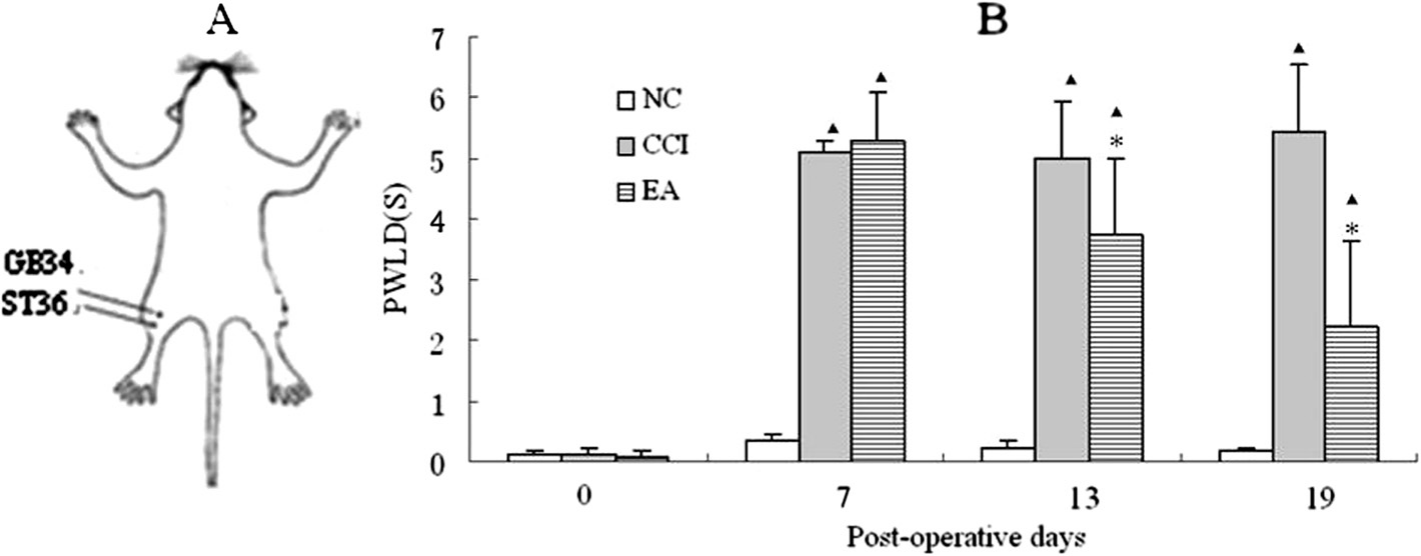
**Schematic locations of the acupoints in rats and effects of EA on pain threshold (A) Schematic locations of the acupoints in rats, (B) Effects of EA on PWLD in rats of the NC, CCI and EA groups (n = 20 per group).** PWLD was recorded before CCI, and on day 7,13 and 19 after CCI. Data are presented as mean ± SD. ▲*P* < 0.05, compared with the NC group; * *P* < 0.05, compared with the CCI group.

According to the theory of traditional Chinese medicine, Zusanli (ST36) and Yanglingquan (GB34) (Figure 1A) are considered the most effective acupoints and are commonly used for treating low back pain in China and other countries [27,28]. In the present study, the animals in the EA group were treated with EA stimulation of bilateral ST36 and GB34 from the 7th day on after CCI. The acupoints were punctured with stainless steel filiform needles (diameter 0.35 mm, length 40 mm, Huatuo; Suzhou Medical Appliance Manufactory, Jiangsu, China) to a depth of approximately 2–3 mm, and stimulated electrically for 30 min using a Han’s EA Stimulator (LH202; Neuroscience Research Center, Peking University, Beijing, China). The stimulating intensity and frequency of EA were 1 mA and 2/15 Hz, respectively. During EA stimulation, the animals were awake and constrained with a special cloth bag. The treatment was administered once daily continuously for 12 consecutive days.

### Protein extraction

After termination of the EA treatment on the 19^th^ day after CCI, all rats in the three groups were deeply anesthetized and killed by decapitation. The hippocampal tissue was removed rapidly on an ice plate, washed with normal saline, and then suspended in ultrasound lysis buffer. The tissue was sonicated for 5 min on ice using a Sonifier750 (Branson Ultrasonics Co., Danbury, CT, USA). After adding 5 mg of RNase and 100 units of RQ1 DNase (Promega, Madison, WI, USA), the tissue lysate was incubated at room temperature for 1 h to fully dissolve the protein, and then centrifuged (45,000 r/min) for 1 h. The supernatant was collected for determining the protein concentration with a Plusone 2-D Quant Kit (Amersham Pharmacia Biotech, Uppsala, Sweden). After qualification of proteins by Bradford method, the supernatants were aliquoted and stored at - 80°C until electrophoresis.

### 2-DE

The first-dimensional iso-electric focusing (IEF) electrophoresis was performed on pre-cast, dry 24 cm immobilized pH gradient strips (pH 3–10), and on a Bio-Rad Isoelectric Focusing System (Bio-Rad, Hercules, CA, USA). Each strip was first hydrated for 2 h with the protein in 300 μL of IEF hydration buffer (7 M urea, 2 M thiourea, 4% CHAPS, 0.5% carrier ampholyte, 40 mM DTT, and 0.002% bromophenol blue). IEF was performed using the following condition: 30 V for 10 h, 200 V for 1 h, 500 V for 1 h, 1000 V for 1 h, and 8000 V for 13 h until IPG strips were removed from the first dimension. The strips were then subjected to a two-step equilibration using equilibration buffer I [6 M urea, 2% (m/V) SDS, 0.375 M Tris–HCl pH (8.8), 30% (V/V) glycerol, and 1% DTT], followed by equilibration buffer II [6 M urea, 2% SDS, 0.375 M Tris–HCl (pH 8.8), 20% glycerol, and 135 mM iodoacetamide] for 15 min respectively just before 2-DE. The second dimension of vertical separation was then performed for approximately 4 h on 12.5% SDS-PAGE at 30 mA/gel. Silver staining of the gels was performed using a silver stain kit (Amersham Pharmacia Biotech).

### Image analysis

The gels were scanned at a resolution of 400 dpi with an Amersham ImageScanner 2D (Amersham Pharmacia Biotech). ImageMaster 2D Platinum 5.0 software was used for spot detection, gel alignment, spot quantification, and log normalized data, followed by calculation of the protein molecular weight (MW) of proteins in synchronizing electrophoresis and the isoelectric point (pI) over the pH range of the IEF gel strips. Images from the three groups were compared. Proteins that had significant differences between the CCI and EA groups were shown in this study. Based on the gel-to-gel variation of reduced error for highly abundant proteins, protein spots that showed at least 2-fold difference [29] in the average spot relative volume between two groups at 95% confidence level (t-test; *p*<0.05) were selected as significantly different. For subsequent mass spectrometry analysis, significant spot coordinates were transferred to a Coomassie stained preparative gel for spot picking.

### Enzymatic digestion of protein spots and mass spectrometry for protein identification

In-gel protein digestion was performed as described previously [30,31]. The digested products were treated with 0.5% TFA/30% ACN solution (25 μL) for 30 min. Peptide mass fingerprinting (PMF) analysis was then performed in a Bruker Reflex™ III MALDI-TOF mass spectrometer (Bruker Daltonik GmnH, Germany) for identification of proteins.

### Protein identification

The monoisotopic peptide masses obtained from MALDI-TOF MS were interpreted utilizing Mascot freeware ( http://www.matrixscience.com). Monoisotopic masses were used to search the *Rattus* databases, allowing a peptide mass accuracy of 150 ppm and one partial cleavage. Two modifications of oxidation of methionine and carbamidomethyl of cysteine were considered. For the unambiguous identified proteins, more than five peptides must be matched, and the sequence coverage must be greater than 15%. The reference descriptions of the searched differential proteins from MASCOT searching were input into online MAS3.0 software ( http://bioinfo.capitalbio.com/mas3/analysis/create) to perform function, networks, and canonical pathway analyses.

### Confirmation of differential proteins by quantitative real-time PCR

After termination of the EA treatment, all rats were deeply anesthetized and killed as above. The hippocampus tissue was removed rapidly on an ice plate and total RNA was extracted using Trizol (Invitrogen, Carlsbad, CA, USA). RNA was reverse transcribed with oligo (dT) and M-MLV reverse transcriptase (Promega, Madison, WI, USA). Primers were designed using Primer 3.0 and synthesized by Sangon (Shanghai, China). Reactions were performed in triplicate using 10 ng of cDNA in a 25 μL reaction that contained 1 μL of each amplification primer (5 μL) and 12.5 μL of real-time PCR Master Mix (Applied Biosystems, Carlsbad, California, USA). Samples were amplified using standard cycling condition, and data were collected and analyzed with SDS 2.3 software. Relative mRNA expression levels were measured. Each assay was evaluated by a comparative method validated by Applied Biosystems with the formula 2^-ΔΔ^ct.

### Confirmation of differential proteins by Western blot analysis

Total protein (30–50 μg) was loaded onto 10% SDS-PAGE gels and transferred onto PVDF membranes (Millipore, Billerica, MA, USA) for Western blot. Primary antibodies and dilutions used were: monoclonal *MBP* antibody (1:200; Millipore), rabbit polyclonal *GOT1* antibody (1:200; Novus Biologicals, Littleton, CO, USA), rabbit polyclonal *PITPNA* antibody (1:500; ProteinTech Group, Chicago, IL, USA), and monoclonal β-actin antibody (1:5000; Millipore). Tissue protein levels were determined by immunoblot of tissue lysates after SDS-PAGE, and were transferred to PVDF membranes. Reactive bands were visualized using the ECL plus Western blotting detection system (Amersham Pharmacia Biotech) after incubating membranes with HRP-linked IgG (Amersham Pharmacia Biotech).

### Statistical analysis

PWLD of the paws of the rats and quantitative real-time PCR data as well as Western blotting data are expressed as mean ± SD, and were analyzed by one-way ANOVA. The least significant difference (LSD) test was performed to compare the differences between every two groups. All *P* values were 2-sided, and *P* < 0.05 was considered significant.

## Results

### Effects of EA on pain threshold

Before ligature of the sciatic nerve, there was no difference in the PWLD between the healthy and the affected hindlimbs in the NC, CCI, and EA groups (*P* > 0.05), suggesting a similar baseline thermal pain threshold in rats in the three groups (Figure 1B). After CCI surgery, the rats showed typical neuropathic behavior reactions, and a significant reduction in the PWL on the side of nerve ligature (*P* < 0.01). The PWLD was increased considerably in both the CCI and EA groups in comparison with the NC group (*P* < 0.05). There were no differences in pain threshold before EA treatment between the CCI and EA groups. With increasing number of EA treatment sessions, there was a gradual decrease in PWLD, with a significant decrease in PWLD in the EA group by 12 treatment sessions compared with the CCI group (*P* < 0.05) (Figure 1B), suggesting an apparent analgesic effect of repeated EA interventions.

### Effects of EA on expression of the differentially expressed proteins

**Figure 2:**
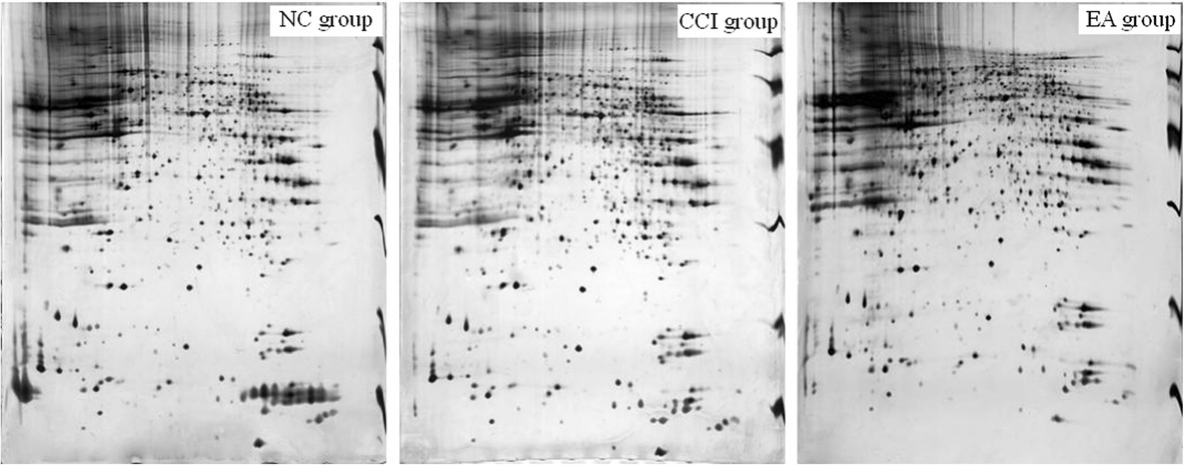
Hippocampal tissue protein profiles obtained with silver stained gels from the three groups.

**Figure 3:**
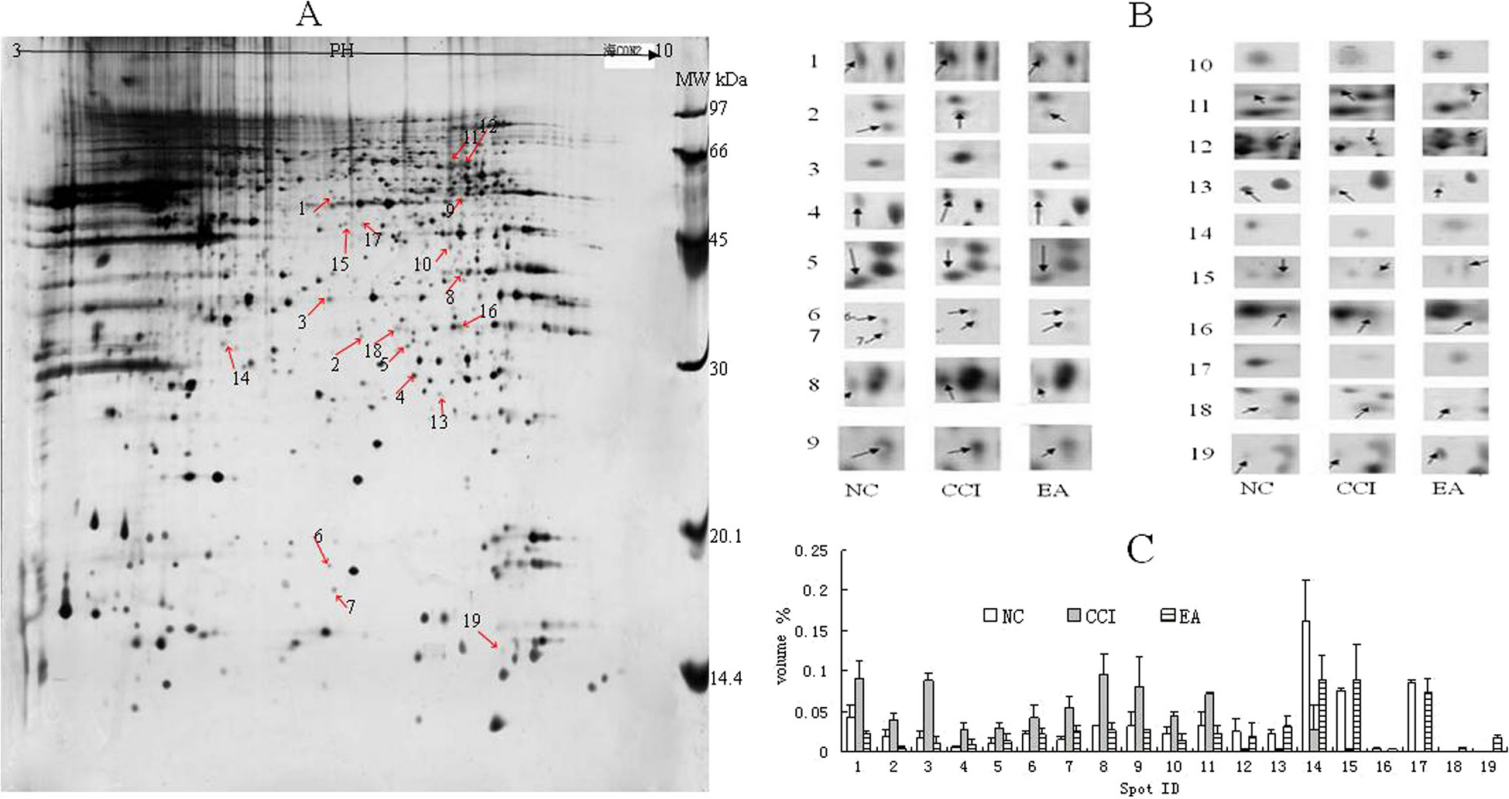
**Expression profiles of the 19 identified differential proteins.(A)** Differential expression profiles of the 19 identified differential protein spots (arrowheads) from pH 3–10 in 2-DE gel templates. **(B)** The excised protein spots from the regions of interest in A. Arrowheads represent protein spots that showed significantly different changes. Spot numbers are consistent with those shown in Table 1. **(C)**. Histograms of the 19 differential proteins in the three groups. Data are presented as mean ± SD (n = 10 per group).

The overview 2-DE silver staining profile from the hippocampus showed that the pI’s of almost all proteins were located between 3 and 10, and there were highly reproducible patterns between the groups (Figure 2). Approximately 800 spots were detected on the ImageMaster 2D Platinum. After matching the maps from the same groups, the average intensities (10 maps per group) of each spot were calculated and compared between the groups. Following 12 sessions of EA, 19 proteins exhibited a differential change in the averaged intensity compared with the CCI group, and were returned to the NC group levels (Figure 3). Of these proteins, 11 were up-regulated and 8 down-regulated after CCI alone.

### Identification of differentially expressed proteins in the hippocampus

**Figure 4:**
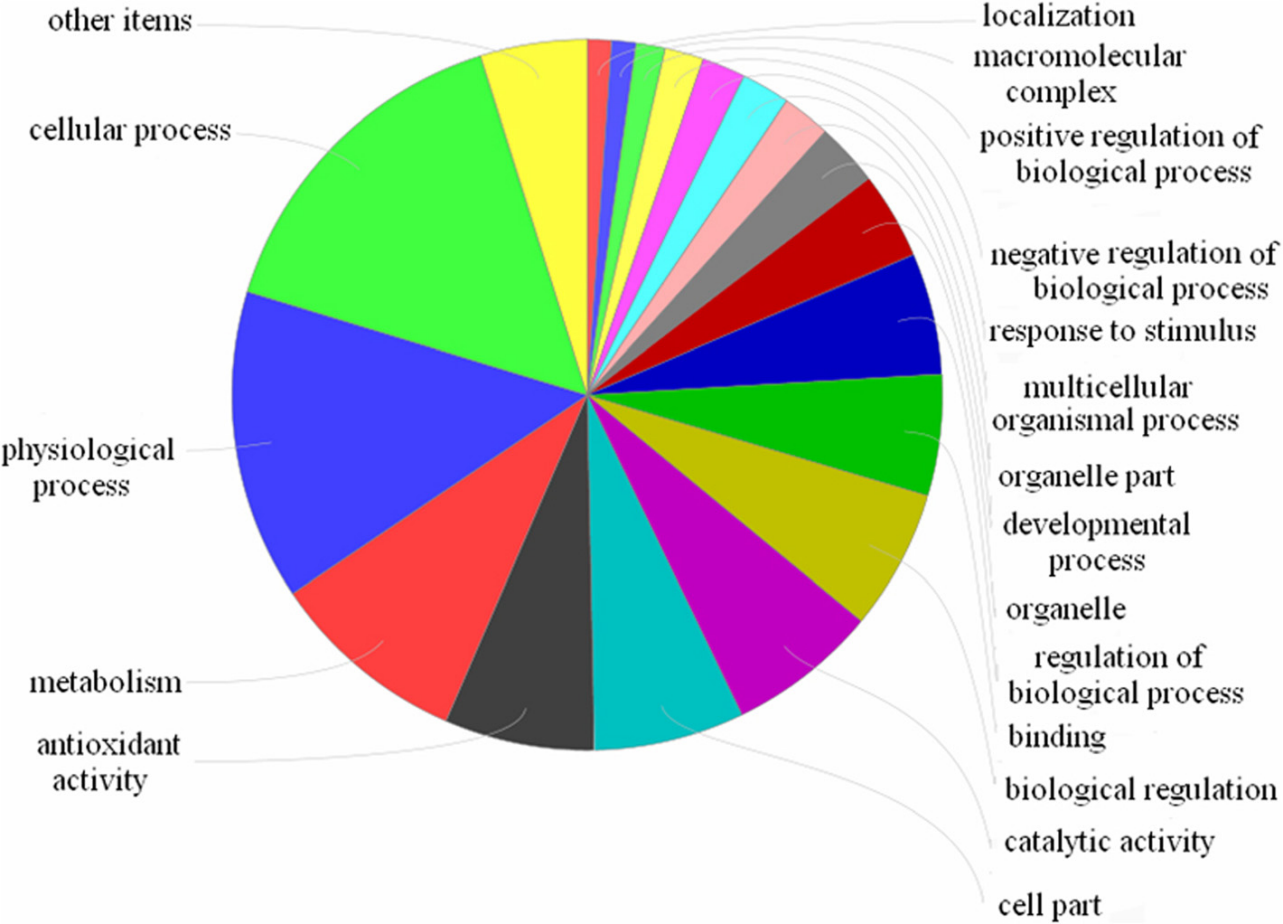
Ontology of differentially expressed proteins.

**Figure 5:**
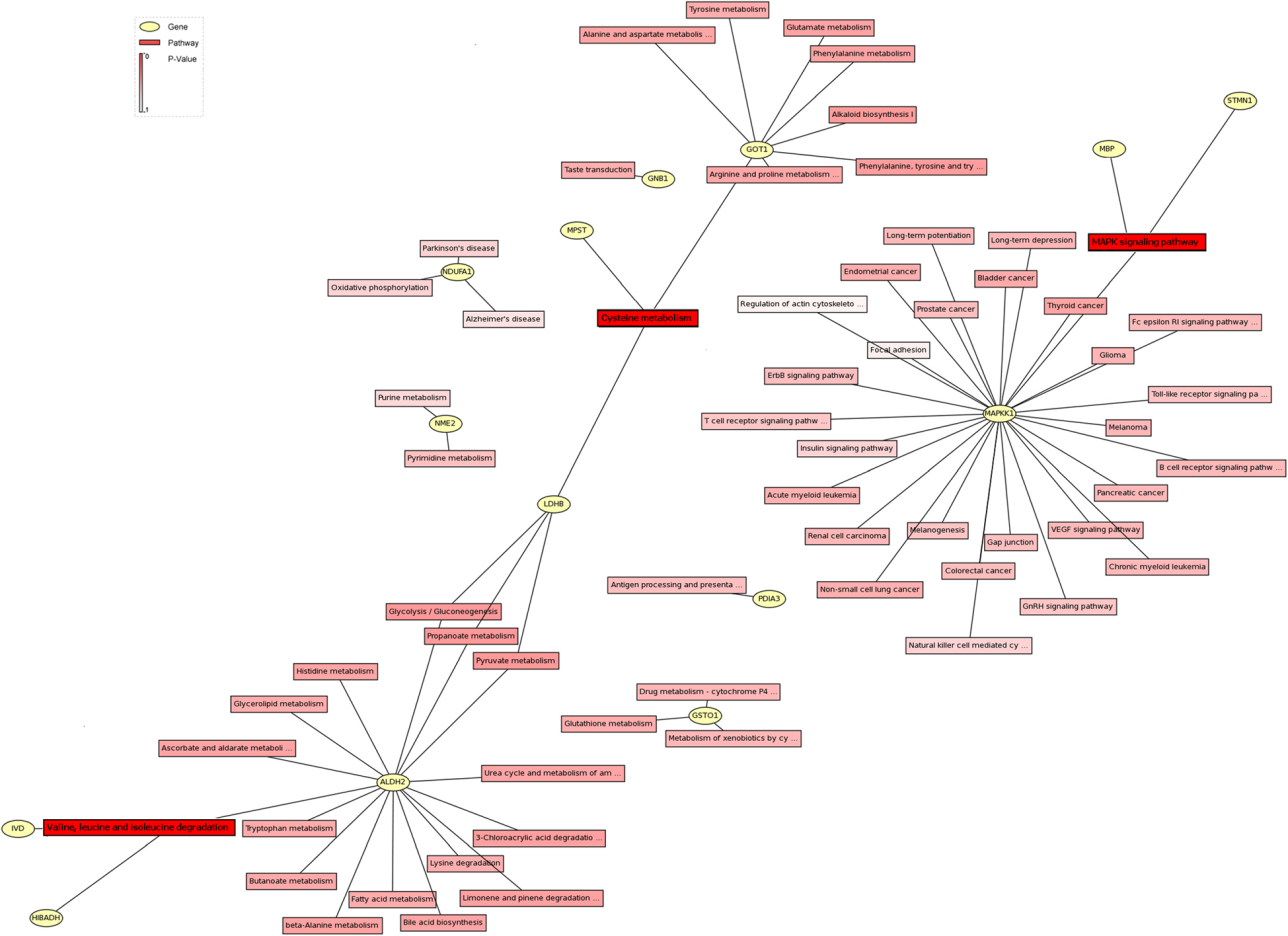
Protein networks of hippocampal differentially expressed proteins.

All the 19 proteins were positively identified in our MS analyses (Table 1). These proteins contributed to over 20 functional clusters (Figure 4), and were comprised of proteins involved in biological processes (46.99%), molecular function (36.14%), and cellular components (16.87%). Kyoto Encyclopedia of Genes and Genomes (KEGG) and genMAPP analysis showed that these proteins were involved in over 60 networks and over 30 pathways (Figure 5). The top-ranked canonical pathways were related to “cysteine metabolism”, “valine, leucine and isoleucine degradation”, and “mitogen-activated protein kinase (MAPK) signaling”.

**Table 1:** List of hippocampal differentially expressed proteins after EA

**Spot ID**	**DB accession**	**Protein name**	**NCBI GI identifier**	**Ratio CCI/NC**	**Ratio EA/CCI**	**Ratio EA/NC**	**Score**	**Protein pI**	**Protein MW**	**Sequence coverage (%)**	**Number of mass values matched**
1	*PDIA3*	protein disulfide isomerase family A, member 3	gi|8393322	2.12	0.25	0.54	261	5.88	57010	51	28
2	*PURA*	purine-rich element binding protein A	gi|293343021	2.13	0.14	0.30	167	6.07	35157	51	13
3	*SIRT2*	sirtuin 2	gi|149056443	4.78	0.13	0.64	137	5.37	43763	44	16
4	*PITPNA*	phosphatidylinositol transfer protein, alpha	gi|8393962	4.65	0.34	1.60	144	5.97	32119	61	22
5	*MPST*	mercaptopyruvate sulfurtransferase	gi|20304123	2.48	0.47	1.16	105	5.88	33205	36	11
6	*STMN1*	stathmin 1	gi|8393696	2.00	0.50	1.01	79	5.76	17278	34	6
7	*NME1*	non-metastatic cells 1	gi|19924089	3.49	0.47	1.63	121	5.96	17296	58	8
8	*NDUFA1*	NADH dehydrogenase (ubiquinone) 1 alpha	gi|170295834	2.93	0.28	0.81	110	7.64	40753	44	15
9	*ALDH2*	aldehyde dehydrogenase 2 family	gi|45737866	2.51	0.34	0.87	117	6.69	56079	39	20
10	*IVD*	isovaleryl-CoA dehydrogenase	gi|6981112	2.01	0.32	0.64	148	8.03	46862	44	18
11	*STIP1*	stress-induced-phosphoprotein 1	gi|20302113	2.14	0.33	0.71	167	6.40	63158	39	19
12	*HNRNPL*	heterogeneous nuclear ribonucleoprotein L	gi|197245939	0.11	7.29	0.77	140	6.70	62572	40	18
13	*GSTO1*	glutathione S-transferase omega 1	gi|12585231	0.11	13.72	1.49	78	6.25	27936	43	9
14	*GNB1*	guanine nucleotide binding protein (G protein), beta polypeptide 1	gi|71089935	0.17	3.35	0.56	74	4.80	28609	39	16
15	*GOT1*	aspartate aminotransferase 1	gi|122065118	0.04	28.61	1.18	166	6.73	46628	36	15
16	*HIBADH*	3-hydroxyisobutyrate dehydrogenase	gi|83977457	0.28	3.81	1.07	64	8.73	35679	24	7
17	*LDHB*	lactate dehydrogenase B	gi|6981146	0.23	3.73	0.87	94	5.70	36874	44	21
18	*MAPKK1*	mitogen-activated protein kinase kinase 1	gi|266566	0.34	4.53	1.56	89	5.89	28267	38	7
19	*MBP*	myelin basic protein	gi|4454311	0.27	5.37	1.42	144	10.9	14184	42	8

### Validation of differentially expressed proteins by real-time PCR and Western blot analysis

**Figure 6:**
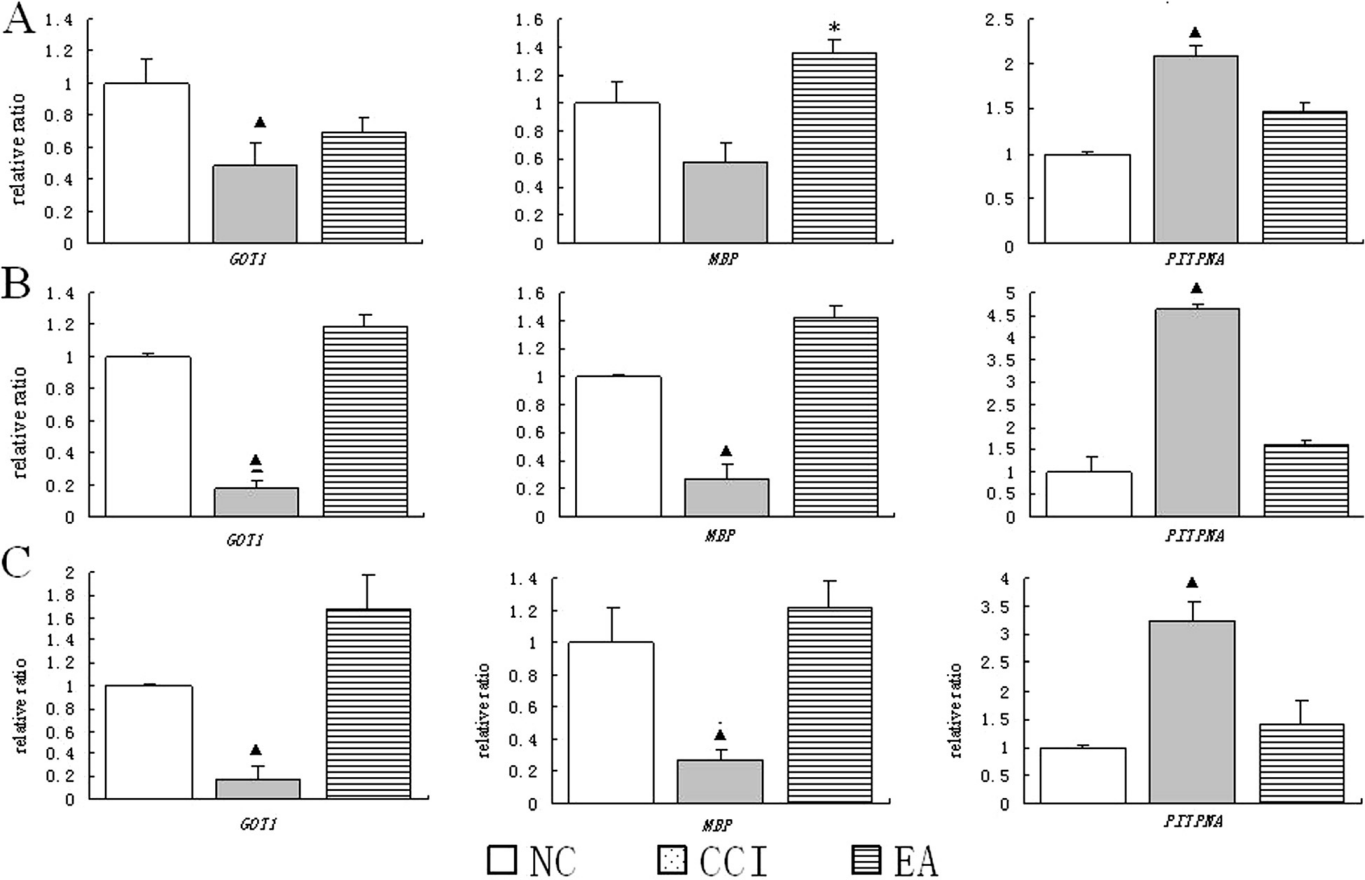
**Expression changes of *PITPNA*, *MBP*, and *GOT1 *in the hippocampus in the NC, CCI, and EA groups were examined by different approaches. A:** by quantitative real-time PCR; **B:** by Western blot; **C:** by 2-D. Data are presented as mean ± SD (n = 10 per group). ▲*P* < 0.05, compared with the NC group; * *P* < 0.05, compared with the CCI group.

To verify the reliability of the proteomics analysis, three hippocampal differential proteins, phosphatidylinositol transfer protein alpha (*PITPNA*), cytoplasmic aspartate aminotransferase (*GOT1*), and myelin basic protein (*MBP*), were selected as representative proteins and subjected to quantitative real-time PCR and Western blot analysis (Figure 6). The mRNA levels of *PITPNA* were significantly up-regulated after CCI in comparison with the NC group (P < 0.05), while there was no change in *GOT1* mRNA expression in the CCI group (*P* > 0.05). Following EA intervention, *PITPNA* and *GOT1* mRNA expression levels were significantly down-regulated in the EA group compared to the CCI group (*P* < 0.05). The expression of *MBP* mRNA in the NC group was comparable to that in CCI group (*P* > 0.05), but was significantly up-regulated in the EA group (*P* < 0.05). The results from Western blot analysis were similar to those from 2-DE.

## Discussion

In the present study, we demonstrated that in CCI-induced neuropathic pain rats, 12 sessions of EA could effectively suppress CCI-induced pain reactions. This result is identical to those reported in our previous studies [26,32]. We also compared global changes of acupuncture-associated proteins in the hippocampus under CCI condition, and found 19 differential expressed proteins that returned to the normal control levels following repeated EA interventions. These differential proteins are involved in physiological, metabolic, and cellular processes, with major pathways including “cysteine metabolism”, “valine, leucine and isoleucine degradation”, and “MAPK signaling”.

There is strong evidence that treatment of sciatica patients using thermal acupuncture can result in a progressive increase in the pain threshold with increasing number of treatment sessions, and an accompanying gradual improvement in symptoms [33]. In this study, we found that stress-induced-phosphoprotein 1 (*STIP1*), which participates in short-term memory formation and long-term memory consolidation in the hippocampus [34,35], was involved in the analgesic effect of EA interventions. In rats with neuropathic pain, Xing et al. [36] demonstrated that EA treatment had a regulatory effect on long-term synaptic plasticity in the spinal dorsal horn. Experimental studies have demonstrated that under pain condition, changes in hippocampal synaptic plasticity are accompanied by changes in learning, memory, and adaptive processes. Interestingly, recent studies have independently shown hippocampal abnormalities in animal models of chronic pain including short-term recognition memory [37], long-term potentiation deficits, abnormal cytokine expression, and impaired enriched-environment neurogenesis [38]. Therefore, Changes of *STIP1* following EA may suggest a direct link between information storage and the analgesic effect of EA.

The functional changes in the hippocampus were accompanied by changes of protein expression, particularly those proteins involving amino acid metabolism. For example, we found that expression of 3-mercaptopyruvate sulfurtransferase (*MPST*), aspartate aminotransferase 1 (*GOT1*), and lactate dehydrogenase B (*LDHB*), which are involved in cysteine metabolism, was decreased after CCI. Among them, *GOT1* is especially important as it also plays a key role in glutamate metabolism. It has been well established that under pain condition, glutamate level in the central never system was increased rapidly, so the decrease of *GOT1* protein level following CCI suggests a shift in the equilibrium of *GOT1* towards glutamate. Our results demonstrated that EA can apparently reverse the decrease of *GOT1*. This may be a critical mechanism of analgesic effect of EA. Chronic pain is also a type of chronic stress. Under chronic stress condition, significant decreases in hippocampal synaptic density and surface density have been reported [38]. Remodeling of the synaptic compartments involves a variety of physiological processes, including enhanced translational activity and related protein synthesis. In the nervous system, neuronal activity is strongly correlated with the levels of amino acid metabolism, and a substantial amount of amino acids are required for changes in protein expression and production of cellular defense mechanisms. Many forms of long-lasting behavioral and synaptic plasticity also require the synthesis of new proteins [39]. Thus, it is feasible that the regulatory activities of many cellular enzymes, especially those in amino acid metabolism found in the present study, can change after CCI.

We also observed a decrease in the expression of valine, leucine, and isoleucine degradation related proteins, isovaleryl-CoA dehydrogenase (*IVD*), 3-hydroxyisobutyrate dehydrogenase (*HIBADH*), and aldehyde dehydrogenase 2 family (*ALDH2*). Karpova et al. [40] reported that hippocampal synaptic activity related to induction of long-term potentiation (LTP) led to a rapid increase in the rate of protein synthesis as well as accelerated protein degradation. Therefore, our study may lend new support to the concept that complementary processes of enhanced protein synthesis and activity-dependent protein degradation is a mechanism underlying control of synaptic protein components [41,42].

Glutathione (GSH), the major endogenous antioxidant produced by cells, has been shown to modulate the activity of N-methyl-D-aspartate receptors (NMDARs) via its reducing effects [43]. In the hippocampus, NMDAR-dependent LTP, a form of synaptic plasticity, is thought to represent a cellular model of memory. The NMDAR is the predominant glutamate receptor involved in hippocampal synaptic plasticity, and is critical for LTP, memory, and learning. Cysteine, a precursor for the formation of glutathione, reverses the L-type calcium channel-dependent LTP seen in the aged animals to NMDAR-dependent LTP [44]. Thus as such, modulating cysteine levels in the hippocampus by acupuncture stimulation may be a therapeutic strategy for treatment of impairments in plasticity and synaptic transmission function in CCI rats.

In response to stress or nerve injury, intracellular pathways that are normally inactive under steady-state condition are activated, while some of the normal pathways may be inhibited or bypassed. In a mouse model of pain induced by subcutaneous injection of formalin, Seo et al. [45] reported that intrathecal and intraperitoneal injection of glutamate or acetic acid resulted in up-regulation of hippocampal phosphorylated Ca^2+^/calmodulin-dependent protein kinase II alpha or phosphorylated extracellular signal-regulated protein expression. In repeated stress stimulation-induced depression rats, EA also increased the number of p-CREB-positive neurons in the hippocampus and restored hippocampal BDNF mRNA expression induced by immobilization stress [46]. These data suggest a role of EA in regulating cellular signal transduction in the hippocampus. We also found evidence of activation of MAPK signal transduction pathway proteins, mitogen-activated protein kinase kinase 1 (*MAPKK1*), stathmin 1 (*STMN1*), and myelin basic protein (*MBP*) following EA [47,48]. The MAPKs are highly conserved across all eukaryotes, and their roles extend beyond the cytoplasm to the nucleus, where they can directly modulate gene transcription. The MAPK signal transduction pathway is also an important signaling event for the induction of hippocampal LTP. Activation of this pathway is necessary for both the transcriptional and translational events underlying long-term memory formation in the hippocampus.

In the nervous system, nerve injury and stress condition can cause oxidative damage to the cytoskeleton and membrane structures. In addition, significant energy production is required for protein expression and cellular defense mechanisms. This is supported by the increased expression of proteins involving antioxidative processes, neuronal integrity, and glycolysis/gluconeogenesis observed in the present study.

The goal of our study was to examine for proteins involved in the analgesic effect of EA. Although there are few comparable studies, using 2-DE-based proteomics, Sung et al. [49] reported differential changes in 36 hypothalamic proteins in a model of rat-tail neuropathic pain, which were all restored to control levels following EA of ST36. Similar to our results, twenty-one of these proteins are involved in biological processes including enzyme metabolism and signal transduction, suggesting common mechanisms of EA-induced analgesia.

The expression of three differential proteins (*PITPNA*, *GOT1*, and *MBP*) was further examined using quantitative real-time PCR and Western blot. Although changes in *PITPNA* and *MBP* mRNA were similar to that for 2-DE, there was no change in *GOT1* mRNA. It is likely that this relates to mRNA-independent post-translational modification, trafficking, and degradation, as a large number of modification sites and domains exist in *GOT1*.

## Conclusions

Taken together, these results demonstrated that repeated EA at ST36 and GB34 produced an analgesic effect against CCI-induced neuropathic pain in rats. A total of 19 differential proteins were found to be involved in this process. Functional analysis showed that EA-mediated neuro-protection may be due to, at least in part, to the normalization of the altered expression of several proteins implicated in cysteine metabolism, valine, leucine, and isoleucine degradation, and MAPK signaling. Further studies are required to examine changes in these proteins over the course of EA treatment and to define their specific functions in regulating the analgesic effect of EA.

## Abbreviations

CCI: Chronic constrictive injury; EA: Electroacupuncture; NC: Normal control; 2-DE: Two-dimensional gel electrophoresis; MALDI-TOF MS: Matrix-assisted laser desorption/ionization time of flight mass spectrometry; PWL: Paw withdrawal latency; PWLD: Difference value of PWL; IEF: First-dimensional iso-electric focusing; MW: Molecular weight; pI: Isoelectric point; MAPK: Mitogen-activated protein kinase.

## Competing interests

The authors declared that they have no competing interests.

## Authors’ contributions

YHG and JLL conceived this study and wrote the manuscript. YHG, SPC, JYW and LNQ carried out the experiment. FYM executed statistical analysis. QLX assisted in matrix-assisted laser desorption/ionization time-of-flight mass spectrometry detection. All authors read and approved the final manuscript.

## Pre-publication history

The pre-publication history for this paper can be accessed here:

http://www.biomedcentral.com/1472-6882/12/241/prepub
